# Dynamic MRI Findings and the mJOA Scale: Establishing Correlations in the Clinical Assessment

**DOI:** 10.1055/s-0045-1811928

**Published:** 2025-11-18

**Authors:** Ricardo André Acácio dos Santos, Raphael de Rezende Pratali, Mariana Demétrio de Sousa Pontes, Carlos Fernando P. S. Herrero

**Affiliations:** 1 Department of Orthopedics and Anesthesiology, Faculdade de Medicina de Ribeirão Preto, Universidade de São Paulo, Ribeirão Preto, SP, Brazil; 2 Department of Orthopedics, Hospital do Servidor Público Estadual, São Paulo, SP, Brazil

**Keywords:** cervical vertebrae, diagnosis, magnetic resonance, diagnóstico, ressonância magnética, vértebras cervicais

## Abstract

**Objective:**

To investigate the correlation between the score on the modified Japanese Orthopaedic Association (mJOA) scale and the dynamic magnetic resonance (DMR) findings in patients diagnosed with degenerative cervical myelopathy (DCM).

**Methods:**

We conducted a retrospective cohort study. All patients underwent a DMR examination of the cervical spine using the same device. The anatomic parameters evaluated were the spinal canal diameter (SCD) and the spinal canal width (SCW). The SCD was measured as the distance between the midpoint of the posterior portion of the intervertebral disc and the anterior margin of the yellow ligament. The SCW was measured as the distance between the anterior and posterior margins of the spinal cord at the exact point used to assess the SCD. The mJOA scale was chosen to assess the functional status. The intra and interobserver reliability of the morphometric parameters of the magnetic resonance imaging (MRI) were calculated using the intraclass correlation coefficient (ICC), and values of *p* < 0.05 were considered statistically significant.

**Results:**

Regarding the intraobserver evaluation of the SCD, an almost perfect agreement was achieved, and the SCW's interobserver evaluation presented a strong agreement. The mJOA score ranged from 6 to 18, and there was a weak, non-statistically significant correlation between the SCD and SCW parameters.

**Conclusion:**

We could not identify a correlation involving the cervical spinal canal measurements obtained from DMR exams and the clinical severity of patients with DCM measured using the mJOA score. Due to the small sample size, these findings should be interpreted with caution.

## Resumo

**Objetivo:**

Investigar a correlação entre a pontuação na escala modificada da Japanese Orthopaedic Association (mJOA) e os achados de ressonância magnética dinâmica (RMD) em pacientes diagnosticados com mielopatia cervical degenerativa (MCD).

**Métodos:**

Conduzimos um estudo de coorte retrospectiva. Todos os pacientes foram submetidos a um exame de RMD da coluna cervical usando o mesmo equipamento. Os parâmetros anatômicos avaliados foram o diâmetro da medula espinhal (DME) e a largura do canal vertebral (LCV). O DME foi medido como a distância entre o ponto médio da porção posterior do disco intervertebral e a margem anterior do ligamento amarelo. A LCV foi medida como a distância entre as margens anterior e posterior da medula espinhal no ponto exato de avaliação do DME. A escala mJOA foi escolhida para avaliação do estado funcional. As confiabilidades intra e interobservador dos parâmetros morfométricos da ressonância magnética (RM) foram calculadas segundo o coeficiente de correlação intraclasse (CCI), e valores de *p* < 0,05 foram considerados estatisticamente significativos.

**Resultados:**

A avaliação intraobservador do DME teve concordância quase perfeita, e a avaliação interobservador da LCV apresentou forte concordância. A pontuação na mJOA variou de 6 a 18. Houve uma correlação fraca e não estatisticamente significativa entre os parâmetros DME e LCV.

**Conclusão:**

Não identificamos uma correlação entre as medidas do canal vertebral cervical obtidas em exames de RMD e a gravidade clínica dos pacientes com MCD medida pela escala mJOA. Devido ao pequeno tamanho da amostra, esses achados devem ser interpretados com cautela.

## Introduction

Degenerative cervical myelopathy (DCM) is the most common source of spinal cord dysfunction in patients older than 60 years of age. 1 The development of DCM is associated with age-related degeneration of the cervical spine and spinal cord compression from the abnormal degenerated tissue. 1 2 Patients with symptomatic DCM may present a combination of motor, sensory and autonomic dysfunctions, and, consequently, reduced quality of life. 1 2

Several questionnaires can be used to assess the severity of the physical disability, the patient's clinical condition, and the effectiveness of the treatment. 3 Among these instruments, the modified Japanese Orthopaedic Association (mJOA) scale has become one of the most frequently used outcome measures to assess functional status in patients with DCM. 4 5 6

Although primarily based on clinical assessment, it is well established that magnetic resonance imaging (MRI) is crucial in the assessment of spinal cord compression and in the diagnosis of DCM. 7 Previous studies 8 9 involving MRI findings have shown that this exam aids in the establishment of the prognosis of the surgical treatment, but not all parameters have been shown to be useful. The factors that have been shown to have an impact include the duration of T2 hyperintensity, congenital stenosis, cervical spondylolisthesis, and T1 hypointensity. 8 9 10 11 12 13 14

Dynamic magnetic resonance (DMR) imaging, which includes cervical spine evaluation during flexion and extension, may better reflect the real-time biomechanical impact of movement on the spinal cord than static MRI alone. 3 8 9 This is particularly relevant in DCM, in which the symptoms often fluctuate with neck position, and transient spinal cord compression may not be fully captured on conventional imaging. 3 8 9 More recently, other studies 3 8 9 aimed to investigate the role of DMR imaging in patients with DCM and demonstrated significant changes in cervical spinal canal measures in a subgroup of patients with DCM; the authors stated that those findings may explain clinical examination findings and, consequently, impact the results of the surgical treatment.

Therefore, to establish a correlation between clinical and magnetic resonance findings, we studied data from a subset of patients enrolled in a prospective research to assess a correlation between the mJOA score and the DMR findings in patients diagnosed with DCM.

## Materials and Methods

### Study Design and Data Collection

Before commencing the study, ethical approval was obtained from the hospital's Ethics Committee and Internal Review Board, and all participants provided informed consent before data collection began. The present research was established as a prospective cohort study aimed at evaluating the reliability of a cervical DMR imaging technique in patients diagnosed with DCM, based on previously-published preliminary findings.

The participants had a clinical diagnosis of DCM confirmed through MRI scans and were then submitted to standard surgical procedures for treatment. The inclusion criteria were patients who filled out the mJOA questionnaire, underwent the DMR exam, and consented to participate in the study. Those who had undergone prior cervical spine surgery or had other orthopedic, neurological, or psychiatric conditions that might influence the clinical outcomes were excluded. Out of 168 potential candidates who filled out the mJOA questionnaire, 18 (14 male and 4 female) patients met the inclusion criteria and had DMR imaging available for subsequent analysis.

### Imaging Acquisition and Evaluation

All enrolled patients underwent a DMR assessment of the cervical spine using a standardized 1.5-Tesla Achieva device manufactured by Philips. The image analysis was carried out using the OsiriX MD, version 7.0, 64-bit software (Primeo SARL), with a consistent zoom level of 300%. Two independent spine surgeons analyzed the images. One of these observers repeated the assessment after 30 days to evaluate the intraobserver reliability, following the same protocol and using the same computer and software for consistency.

### Anatomical Measurements

**Fig. 1:**
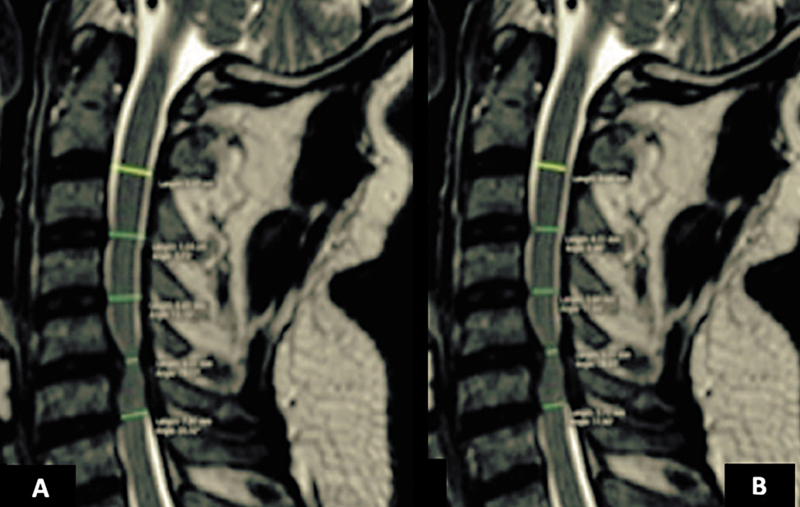
T2-weighted sagittal cervical magnetic resonance imaging scan. ( **A** ) Spinal cord width. ( **B** ) Spinal cord diameter.

The anatomical parameters assessed included the spinal cord diameter (SCD) and the spinal canal width (SCW). The SCW was calculated by measuring the distance between the midpoint of the posterior part of the intervertebral disc and the anterior limit of the yellow ligament ( Fig. 1A ). The SCD was determined as the distance from the anterior to the posterior margins of the spinal cord at the same point where the SCW was measured ( Fig. 1B ). All measurements were linear and recorded in millimeters (±1.0 mm), captured from midline images of the T2-weighted sequences in the sagittal plane under neutral position and in flexion and extension at the disc spaces from C2-C3 to C6-C7.

### Clinical Evaluation

The mJOA scale was chosen to assess the functional status; it is an 18-point DCM scale that separately scores upper (5 points) and lower (7 points) extremity motor function, sensation (3 points), and sphincter control (3 points). 5

### Statistical Analysis

The statistical analysis was performed using the STATA13 (StataCorp LLC) software. The intra- and interobserver reliability of the morphometric parameters of the DMR were calculated using the intraclass correlation coefficient (ICC), with a 95%CI. Values of the ICC ranging from 0.00 to 0.20 were considered poor agreement, from 0.21 to 0.40, fair agreement, from 0.41 to 0.60, moderate agreement, from 0.61 to 0.80, strong agreement, and from 0.81 to 1.00, almost perfect agreement. The statistical correlation analysis considered the severity of the DCM, represented by the mJOA score, and the dynamic measures using the Spearman rank correlation test. Values of *p* < 0.05 were considered statistically significant.

## Results

### Subjects

A total of 18 eligible patients completed the DMR protocol, fulfilled the inclusion criteria and were analyzed in the current study. There were 14 men and four women, with a mean age of 60 (range: 37–76) years.

### Interobserver Reliability

Regarding the SCD evaluation, the mean ICC values of all disc levels for interobserver reliability were of 0.90 in the neutral position, and of 0.92 in flexion and extension ( Table 1 ). The SCW interobserver reliability had mean ICC values of 0.80, 0.88, and 0.87 for the neutral, flexion, and extension positions respectively ( Table 1 ).

**Table 1:** Interobserver reliability regarding the spinal canal width and the spinal cord diameter according to the intraclass correlation coefficient

	Flexion (95%CI)	Neutral position (95%CI)	Extension (95%CI)
	**Spinal canal width**		
**C2** – **C3**	0.94 (0.86–0.98)	0.95 (0.87–0.98)	0.97 (0.92–0.98)
**C3** – **C4**	0.89 (0.73–0.96)	0.98 (0.95–0.99)	0.95 (0.88–0.98)
**C4** – **C5**	0.87 (0.66–0.95)	0.94 (0.86–0.98)	0.89 (0.70–0.95)
**C5** – **C6**	0.93 (0.83–0.97)	0.93 (0.81–0.97)	0.92 (0.78–0.98)
**C6** – **C7**	0.90 (0.75–0.96)	0.83 (0.56–0.93)	0.91 (0.78–0.96)
	**Spinal cord diameter**		
**C2** – **C3**	0.84 (0.59–0.94)	0.96 (0.91–0.98)	0.94 (0.86–0.98)
**C3** – **C4**	0.92 (0.80–0.97)	0.95 (0.88–0.98)	0.94 (0.84–0.97)
**C4** – **C5**	0.84 (0.57–0.94)	0.91 (077.–0.96)	0.95 (0.89–0.98)
**C5** – **C6**	0.95 (0.87–0.98)	0.95 (0.87–0.98)	0.90 (0.75–0.96)
**C6** – **C7**	0.73 (0.29–0.90)	0.82 (0.54–0.93)	0.82 (0.52–0.93)

### Intraobserver Reliability

Regarding the intraobserver reliability of the SCD values, the neutral position had a mean ICC of 0.97, the flexion position, of 0.96, and the extension position, of 0.97, with all disc levels having an ICC higher than 0.90 in every position. For the SCW evaluation, the results showed that the neutral position had a mean ICC of 0.94, with the flexion and extension positions having mean ICC values of 0.87 and 0.94 respectively. The ICC values for each disc level were higher than 0.8 for every position in all disc levels. Table 2 presents the complete description of ICC values for each position and disc level.

**Table 2:** Intraobserver reliability regarding the spinal canal width and the spinal cord diameter according to the intraclass correlation coefficient

	Flexion (95%CI)	Neutral position (95%CI)	Extension (95%CI)
	**Spinal canal width**		
**C2** – **C3**	0.96 (0.91–0.98)	0.98 (0.97–0.99)	0.99 (0.97–0.99)
**C3** – **C4**	0.98 (0.95–0.99)	0.99 (0.98–0.99)	0.98 (0.97–0.99)
**C4** – **C5**	0.94 (0.86–0.98)	0.96 (0.91–0.98)	0.99 (0.99–0.99)
**C5** – **C6**	0.98 (0.96–0.99)	0.98 (0.94–0.99)	0.93 (0.83–0.97)
	**Spinal cord diameter**		
**C2** – **C3**	0.84 (0.59–0.94)	0.96 (0.91–0.98)	0.94 (0.86–0.98)
**C3** – **C4**	0.92 (0.80–0.97)	0.95 (0.88–0.98)	0.94 (0.84–0.97)
**C4** – **C5**	0.84 (0.57–0.94)	0.91 (0.77–0.96)	0.95 (0.89–0.98)
**C5** – **C6**	0.95 (0.87–0.98)	0.95 (0.87–0.98)	0.90 (0.75–0.96)

### Correlation between DMR findings and mJOA Score

**Fig. 2:**
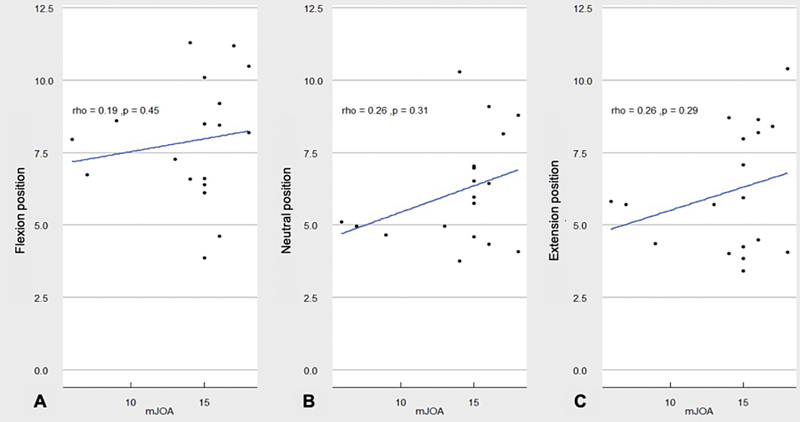
Correlation between the score on the modified Japanese Orthopaedic Association (mJOA) scale and spinal cord diameter in flexion ( **A** ), neutral position ( **B** ), and in extension ( **C** ).

The mJOA score ranged from 6 to 18 (median: 15) points. The Spearman classification test, which was used to assess the correlation between the results obtained from the mJOA score evaluation and the results of the SCD, showed a weak positive correlation in the flexion position (0.191), with *p* = 0.448 ( Fig. 2A ), a weak positive monotonic relationship in the neutral position (0.255), with *p* = 0.307 ( Fig. 2B ), and a weak positive correlation in the extension position (0.265), with *p* = 0.288 ( Fig. 2C ).

**Fig. 3:**
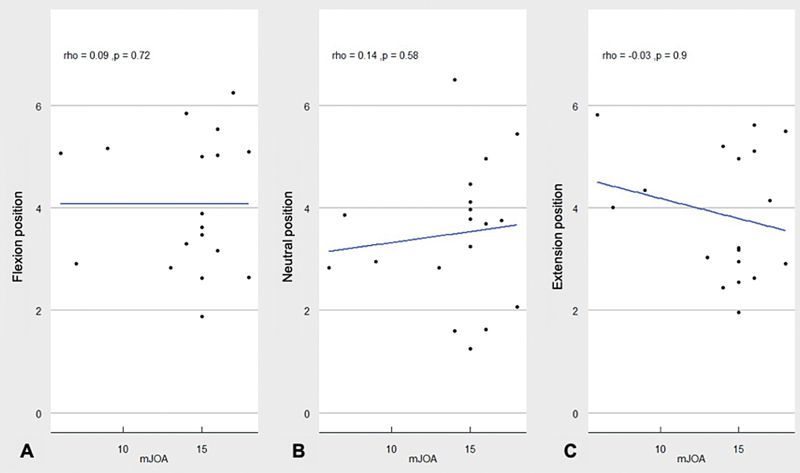
Correlation between the score on the modified Japanese Orthopaedic Association (mJOA) scale and spinal canal width in flexion ( **A** ), neutral position ( **B** ), and in extension ( **C** ).

When evaluating the correlation between the mJOA score and the SCW results, the Spearman rank test evidenced a very weak positive correlation (0.089), with *p* = 0.724 in the flexion position ( Fig. 3A ), a weak positive correlation (0.14), with *p* = 0.507 in the neutral position ( Fig. 3B ), and a weak negative correlation (−0.032), with *p* = 0.898 in the extension position ( Fig. 3C ).

## Discussion

In the current study, we found no clear association between the DMR imaging findings and the mJOA questionnaire results in patients with DCM. Degenerative cervical myelopathy is the most common cause of spinal cord dysfunction, characterized by reduced spinal canal measurements due to degenerative changes exacerbated by cervical spine movements, which can be observed through cervical DMR imaging. 15 16 Although the literature 3 4 5 6 7 8 9 10 11 12 13 14 15 16 17 presents differing views on the link between MRI findings and clinical features of DCM, the present study is, to the best of our knowledge, the first to measure morphometric parameters of the cervical spinal canal using DMR exams and correlate them with mJOA questionnaire results.

Magnetic resonance imaging is considered an important prognostic tool for DCM patients, as evidence to support this concept has been published and indicates that reduction in the cross-sectional area of the cervical spinal canal and signal changes in the spinal cord of DCM patients are significantly associated with the degree of neurological severity, the prevalence of specific clinical manifestations, and potential for neurological recovery. 18 19 20 In patients with DCM, clinical manifestation results from degenerative anatomical alterations that cause compression of the spinal canal, and MRI scans of the cervical spine provide static images of spinal cord compression. 17 18 19 However, it has been well demonstrated 15 that dynamic compression resulting from hypermobility and instability of the cervical joint can aggravate an existing spinal canal narrowing. Thus, DMR study data demonstrated that the different positions (neutral and in flexion and extension) influence the measurements of the diameter of the cervical spinal canal and the spinal cord. 9

The present research provided data on the cervical spinal canal and cord morphometric parameters in patients with DCM based on MRI scans acquired in the neutral, flexion, and extension positions. We evaluated inter- and intraobserver reliability of the cervical spinal morphometric parameters acquired based on the DMR protocol adopted at our institution. 21 The measurements of all dynamic parameters presented at least substantial agreement for inter and intraobserver reliability. Likewise, Yu et al. 22 showed that DMR has been proven to be a suitable method to identify instability in the cervical spine. Other authors 15 showed that patients with cervical spine instability had worse mJOA and Nurick scores and worse electrophysiological findings signals than those without instability. Additionally, Nigro et al. 16 suggested that, using DMR imaging, surgeons can identify more findings associated with DCM and worsening compression than with traditional cervical MRI.

The mJOA scale, a helpful tool in assessing DCM, has been evaluated for reliability and validity. 5 6 There is no ideal clinical criterion to evaluate patients with symptoms of spinal cord compression. The mJOA scale was chosen because it is the most widely used criterion in clinical studies and is easy to apply. Therefore, as the correlation between findings on cervical DMR and disease severity remains controversial, the present study tried to establish a correlation between the cervical DMR exam findings and the score on the mJOA scale. 5 6 However, our results obtained from the correlation of the mJOA scores with the dynamic morphometric parameters of the cervical spine did not show statistically significant results. Moreover, although the correlation did not reach a significance level, which may have occurred due to sample-size issues, none of the cervical positions showed correlations higher than the others, which might suggest that there is not a clear straight line between spinal canal diameter and myelopathy, but rather the most significant factor in predicting changes in clinical conditions could be the changes in the signal intensity of the spinal cord, as seen in the diffusion tensor imaging (DTI). 23 The absence of a positive correlation between the mJOA score and the SCW in extension can also be explained by the fact that the group of patients studied presented high initial and mJOA levels.

Several limitations deserve consideration when interpreting the findings of the current study. The relatively small sample size and the homogeneity in the severity of clinical findings, as represented by the limited variation in mJOA scores, could have introduced a sampling bias and influenced our results. A larger patient cohort, with a wider range of mJOA scores, may reveal statistically significant correlations between cervical DMR findings and clinical outcomes. In addition, the present study did not include an evaluation of spinal cord MRI signal changes, which may serve as indicators of more severe clinical findings and, consequently, worse mJOA scores. Moreover, the exclusion of spinal cord signal abnormalities—such as T2 hyperintensity and DTI-based metrics—represents a significant limitation of our analysis, as these variables may better capture underlying neural compromise and explain functional deficits more accurately than morphometric measurements alone. From a clinical standpoint, our findings suggest that DMR morphometric measurements alone may not be sufficient to estimate functional impairment in DCM patients, highlighting the need for a more comprehensive imaging approach that includes spinal cord signal assessment. This insight reinforces the importance of a multimodal evaluation in the diagnosis and prognostication of DCM. Despite these limitations, the current study presents an innovative way to correlate findings on DMR with the mJOA score, representing a model for future studies with larger and more heterogeneous patient populations, as well as the inclusion of spinal cord signal intensity metrics, to further elucidate the complex relationship between dynamic spinal canal morphology and clinical presentation in DCM.

## Conclusion

We found results that conflicted with those of previously-published studies, as it was not possible to identify a correlation between measurements of the cervical spinal canal in the DMR and the clinical severity of patients with DCM measured using the mJOA scale. Thus, our results reinforce the need for additional research on DMR, since the inherent movements of the cervical spine are associated with the pathophysiology of DCM.
